# Relationship between body mass index and bone mineral density in HIV-infected patients referred for DXA

**DOI:** 10.7448/IAS.17.4.19569

**Published:** 2014-11-02

**Authors:** Carmela Pinnetti, Lupi Federico, Patrizia Lorenzini, Chiappetta Domenico, Bellagamba Rita, Loiacono Laura, Mauro Zaccarelli, Stefania Cicalini, Raffaella Libertone, Alberto Giannetti, Silvia Mosti, Elisa Busi Rizzi, Andrea Antinori, Adriana Ammassari

**Affiliations:** 1 National Institute for Infectious Disease, Clinical Department, Rome, Italy; 2 National Institute for Infectious Disease, Radiological Department, Rome, Italy

## Abstract

**Introduction:**

Reduced bone mass density (BMD) is a frequent observation in HIV-infected persons. Relationship between body mass index (BMI), weight, height and BMD was reported for many populations. In particular, BMI has been found to be inversely related to the risk of osteoporosis.

**Methods:**

This is a cross-sectional, monocentric study where all HIV-infected patients referred to first DXA scan in clinical routine during 2010–2013 were included. Osteopenia and osteoporosis were defined by T- score <−1 and <−2.5, respectively. Patients were categorized according to WHO BMI classification: underweight <18.5 kg/m^2^; normal weight 18.5–24.9 kg/m^2^; over weight 25–29.9 kg/m^2^; obese >30 kg/m^2^. Statistical analysis was carried using logistic regression.

**Results:**

A total of 918 patients were included: median age 49 years (IQR, 44–55); 59.4% male; 93% Caucasian. Median anthrometric characteristics were: 68 kg (IQR, 59–78); 1.7 m (IQR, 1.6–1.75); 23.5 kg/m^2^ (IQR, 21.4–26.2). Underweight was found in 5%, normal weight in 61%, overweight in 26% and obesity in 8% of patients. According to T-scores, 110 (11.2%) patients were osteoporotic and 502 (54.7%) had osteopenia. In the femoral neck area, the prevalence of osteoporosis was slightly lower (5.7%) than lumbar spine site (9.2%). Agreements between sites of T-scores for the diagnosis of osteoporosis were 26 and 172 and 346 for osteopenia and normal BMD values, respectively. T-scores at femoral neck or lumbar spine positively correlated with BMI (p<0.001) (Figure 1). Among predictors of osteopenia/osteoporosis, univariable analysis showed: older age (p<0.0001); lower weight (p<0.0001); increasing height (p<0.002). Patients underweight had a higher risk of osteopenia (p=0.02) as well as of osteoporosis (p=0.003). Patients with BMI above normal had a reduced risk of low BMD (osteopenia p<0.0001; osteoporosis p<0.03). Controlling for calendar year, gender, ethnicity, and age, BMI was confirmed as risk factor if below normal (AdjOR of osteopenia 2.42 [95% CI 1.16–5.07] p=0.02; AdjOR of osteoporosis 3.22 [95% CI 1.60–6.49] p=0.001).

**Conclusions:**

Our findings indicate that almost 66% of HIV-infected patients have subnormal bone mass. Further, as in other patient populations, in the HIV infection also low BMI is an important risk factor for osteopenia/osteoporosis. This finding highlights the compelling need for standardized screening actions, particularly in patients weighting below normal.

**Figure 1:**
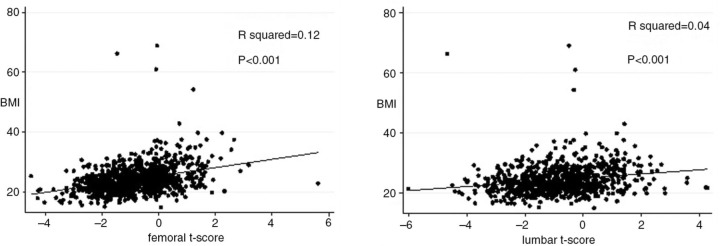
Scatterplot of BMI by frmoral and lumber t-score. Linear regression line was fitted.

